# The Efficacy of Teriparatide in Improving Fracture Healing in Hip Fractures: A Systematic Review and Meta-Analysis

**DOI:** 10.1155/2020/5914502

**Published:** 2020-08-20

**Authors:** Shuang Han, Shi-Ming Wen, Qin-Peng Zhao, Hai Huang, Hu Wang, Yu-Xuan Cong, Kun Shang, Chao Ke, Yan Zhuang, Bin-Fei Zhang

**Affiliations:** ^1^Department of Orthopedic Trauma, Honghui Hospital, Xi'an Jiaotong University, Beilin District, Xi'an, Shaanxi Province, China; ^2^Department of Emergency, Honghui Hospital, Xi'an Jiaotong University, Beilin District, Xi'an, Shaanxi Province, China

## Abstract

**Background:**

This systematic review and meta-analysis assessed the role of teriparatide in improving hip fracture healing and function to provide a clinical guide.

**Methods:**

The systematic literature review identified randomized controlled trials (RCTs) and controlled studies evaluating teriparatide for elderly hip fractures. A meta-analysis was performed using RevMan version 5.3.

**Results:**

This study included two RCTs and four retrospective studies comprising 607 patients, with 269 and 338 patients in the teriparatide and control groups, respectively. The quality of these six studies was moderate. Compared to the control group, teriparatide reduced the time to union (weighted mean difference (WMD) = −1.95; 95% confidence interval (CI): -3.23–-0.68; *P* = 0.003) but did not improve the rate of fracture union at 3 months (odds ratio (OR) = 1.46; 95% CI: 0.50–4.24; *P* = 0.49) or 6 months (OR = 0.89; 95% CI: 0.44–1.81; *P* = 0.75). In addition, teriparatide did not decrease the complications, need for reoperation, mortality, rate of deformity after fracture healing, and subsequent fracture or improve hip function.

**Conclusions:**

The current limited evidence did not support that teriparatide improves fracture healing in hip fractures, due to study heterogeneity and various sources of biases. Further high-quality, large-sample trials are needed. This trial is registered with PROSPERO with registration number CRD42020152205.

## 1. Introduction

The estimated annual numbers of hip fractures worldwide are as high as 4.6 million by 2025 and 6.26 million by 2050 [1, 2]. Fractures at this site often contribute to high mortality and adverse outcomes in the geriatric population. During recovery from fracture, most patients experience fracture-reduced mobility and impaired ability to perform routine daily activities, with a large proportion failing to regain their prefracture functional level after 1 year [3].

Most hip fractures are managed surgically with open reduction and internal fixation (ORIF) or arthroplasty [4, 5]; otherwise, patients are managed without surgery [6]. Regardless of the treatment protocol, the goals are pain relief, improved mobilization, and prevention of complications associated with comorbidities after fracture [7]. After primary management, patients should be followed up to assess fracture healing.

In general, slow recovery after hip fracture is associated with negative consequences [8]; thus, there is a medical need to improve healing and functional recovery after hip fracture by rapidly improving hip function without compromising functional outcomes [9]. Thus, various management methods have been considered supplementary treatment. While locally applied pharmacologic therapies have been approved in some countries to accelerate bone healing, the use of systemic agents for this purpose is controversial [10].

Teriparatide (recombinant human parathyroid hormone (PTH) (1–34)) is approved for the treatment of osteoporosis in patients at high fracture risk [11]. Treatment of postmenopausal osteoporosis with teriparatide could decrease the risk of nonvertebral fractures by increasing femoral and total-body bone mineral density [11]. Teriparatide also enhanced bone healing in animal models [12, 13]. Some surgeons have assessed the role of teriparatide in healing in hip fractures [14, 15]; moreover, studies have reported that teriparatide improved radiographic signs of fracture healing [16] and early clinical outcomes [15] in hip fractures but did not decrease the risk of revision surgery or complications [16]. However, other studies have reported negative outcomes [17]. Thus, the effect of teriparatide on fracture healing remains uncertain. Further studies are needed to demonstrate the effects of teriparatide therapy in patients with hip fracture.

Therefore, this systematic review and meta-analysis assessed the role of teriparatide in improving hip fracture healing and function to provide clinical guidance.

## 2. Methods

### 2.1. Inclusion and Exclusion Criteria

The inclusion criteria were as follows: (1) randomized controlled trial (RCT) or controlled studies, (2) participants with hip fractures (femoral neck and intertrochanteric fracture), (3) patients receiving initial surgical treatment before teriparatide or placebo or control administration, and (4) reported outcomes including fracture healing, function, and adverse events in follow-up.

The exclusion criteria were case series without comparison groups and studies not reporting on the outcomes of interest.

### 2.2. Literature Search

We searched the MEDLINE, Embase, and Cochrane Library databases using the keywords teriparatide, parathyroid hormone, PTH, Forsteo, hip fracture, intertrochanteric fracture, trochanteric fracture, pertrochanteric fractures, and femoral neck fracture. The retrieval dates included the time from database creation to Feb 2020. There were no limitations in the search process.

### 2.3. Outcome Measures

The primary endpoints were the time to union and rate of fracture union; the secondary endpoints were reoperation, mortality, deformity, complications, subsequent fracture, and hip function. Fracture union was evaluated by X-ray. Radiological union was defined as bridging at the fracture site by a callus or a cortical continuity involving at least two cortices in the hip using the anteroposterior and lateral views of the femur. The time to union was the time of postoperation to the time of fracture union, and the radiograph should be examined monthly from postoperative until the fracture had healed. The complications mainly included deep and superficial wound infection, delayed union, nonunion, implant failure, reduction loss, and screw migration.

### 2.4. Data Extraction and Quality Evaluation

We screened all titles of the retrieved articles and removed duplicates. After eliminating irrelevant articles, the summaries of the remaining articles were assessed to confirm the adequacy of information. This was followed by reading the full texts. Two investigators resolved disagreements through discussion, and unresolved disagreements were discussed with a third investigator. We assessed the RCTs using the *Cochrane Library Handbook* 5.1 for adequate sequence generation, allocation concealment, blinding, incomplete outcome data, selective reporting bias, and other bias. The Newcastle-Ottawa Scale (NOS) was used as the tool to assess the nonrandomized studies [18].

### 2.5. Statistical Methods

Odds ratios (ORs) and weighted mean differences (WMDs) were used to assess the effect sizes with 95% confidence intervals (95% CIs). The statistical methods included the Mantel-Haenszel (M-H) and inverse variance (I-V) tests. We assessed heterogeneity with *I*^2^ statistics. During quantitative synthesis, a fixed-effects model was employed for low heterogeneity (*I*^2^ < 50%, *P* > 0.1). When heterogeneity was high (*I*^2^ > 50%, *P* < 0.1), we first explored the possible sources of heterogeneity or used a random-effects model. *P* < 0.05 was considered a statistically significant difference. RevMan version 5.3 (The Cochrane Collaboration, Copenhagen, Denmark) was used to perform the analyses [19].

## 3. Results

### 3.1. Included Studies

**Figure 1:**
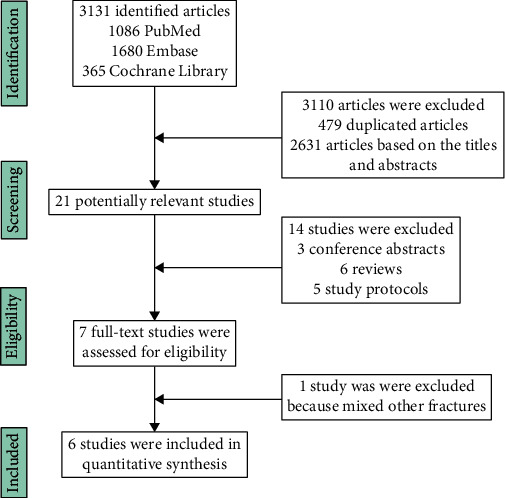
Flowchart of the studies included in the meta-analysis.

Of 3131 potentially eligible articles, most were excluded due to duplications and lack of relevance. Finally, six studies [14–17, 20, 21], including two RCTs [16, 21] and four retrospective studies [14, 15, 17, 20], satisfied the inclusion criteria.  Figure 1 shows the flow of studies through the trial.

### 3.2. Characteristics and Quality Evaluation of the Included Studies

The six studies comprised a total of 607 patients, including 269 and 338 in the teriparatide and control groups, respectively. The sample sizes in each study ranged from 29 [21] to 159 [16]. One study included femoral neck fracture [16]; the remaining studies [14, 15, 17, 20, 21] included intertrochanteric fracture. For primary treatment, the studies used ORIF and intramedullary and extramedullary implants, for the treatment of hip fracture. The dose and frequency of teriparatide use reported in the studies ranged from 20 *μ*g once daily to 56.5 *μ*g weekly. The treatment duration varied from 6 weeks to 18 months. In two RCTs [16, 21], the control group was placebo (identical device) [16] or standard control [21]. In the retrospective studies [14, 15, 17, 20], the control group did not receive teriparatide. The studies performed follow-up ranging from 3 to 40.1 months. Most studies focused on fracture union, reoperation, pain, mortality, and complications, as shown in Table 1.

The quality of the studies was assessed according to the referenced criteria. In the study by Bhandari et al. [16], the random sequence generation, which used a table-based randomization scheme with a block of two, had a low risk of bias. The allocation concealment was unclear. The single-blind method applied for the patients had a low risk of bias. In the study by Chesser et al. [21], random sequence generation by computer-generated blocks of ten also had a low risk of bias; similarly, the allocation concealment had a low risk of bias since sealed envelopes were used. However, the study used blinded outcome assessment rather than blinding during the procedure, which had a high risk of bias. These two studies had low risks of bias related to incomplete outcome data, selective reporting bias, and other bias. Thus, the quality of the two RCTs was moderate. The NOS was used to assess the quality of the controlled studies included in this study; the detailed assessment is shown in Table 2. The total scores were mainly 5 or 6, corresponding to moderate quality. Overall, the quality of the six included studies was moderate.

### 3.3. Primary Endpoints

#### 3.3.1. Time to Union and Rate of Fracture Union

**Figure 2:**
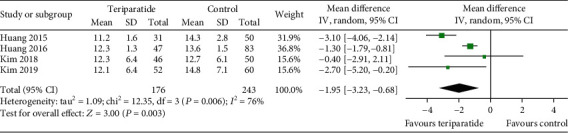
Forest plot comparing time to union in the teriparatide and control groups.

Four studies compared the time to union between the teriparatide and control groups [14, 15, 17, 20]. As shown in Figure 2, the *I*^2^ value for heterogeneity was 76% (*P* = 0.006). After excluding the possibility of clinical heterogeneity, a random-effects model was applied. The time to union in the teriparatide group was shorter than that in the control group (WMD = −1.95; 95% CI: -3.23–-0.68; *P* = 0.003). The results remained stable in a sensitivity analysis that excluded studies individually.

**Figure 3:**
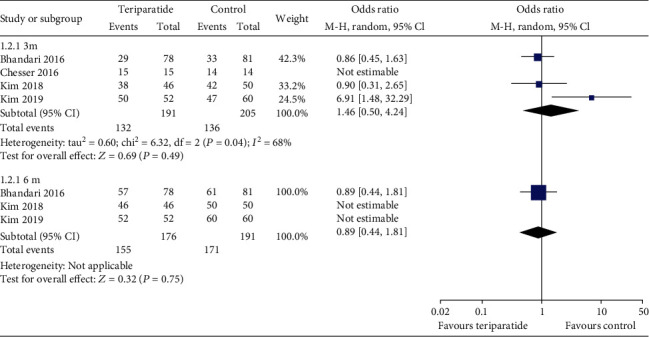
Forest plot comparing the rates of fracture union between the teriparatide and control groups.

Four studies evaluated the rate of fracture union at 3 and 6 months [16, 17, 20, 21]. As shown in Figure 3, the aggregate results of these studies were divided into two subgroups according to the study design. Since the *I*^2^ value for heterogeneity at 3 months was 68% (*P* = 0.04), the random-effects model was used. There were no significant differences in the rates of fracture union at 3 months (OR = 1.46; 95% CI: 0.50–4.24; *P* = 0.49) and 6 months (OR = 0.89; 95% CI: 0.44–1.81; *P* = 0.75) between the two groups. The results remained stable in a sensitivity analysis that excluded studies individually.

### 3.4. Secondary Endpoints

#### 3.4.1. Reoperation

**Figure 4:**
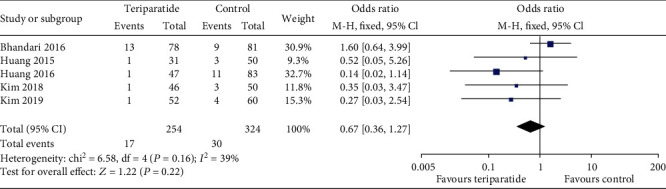
Forest plot comparing reoperation in the teriparatide and control groups.

Five studies assessed reoperation [14–17, 20]. As shown in Figure 4, the aggregate results showed an *I*^2^ value for heterogeneity of 39% (*P* = 0.16); thus, the fixed-effects model was used. There was no significant difference in the rate of reoperation (OR = 0.67; 95% CI: 0.36–1.27; *P* = 0.22) between the two groups. The results remained stable in a sensitivity analysis that excluded studies individually.

#### 3.4.2. Mortality

**Figure 5:**
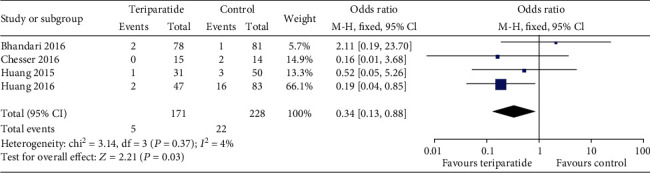
Forest plot comparing mortality in the teriparatide and control groups.

Four of the included studies assessed mortality [14–16, 21]. As shown in Figure 5, the aggregate results showed an *I*^2^ value for heterogeneity of 4% (*P* = 0.38); thus, the fixed-effects model was used. A significant difference in mortality was observed between the groups, in which mortality in the teriparatide group was lower than that in the control group (OR = 0.34; 95% CI: 0.13–0.88; *P* = 0.03). The results of the random-effects model showed no significant difference in mortality (OR = 0.37; 95% CI: 0.12–1.09; *P* = 0.07).

#### 3.4.3. Deformity

**Figure 6:**
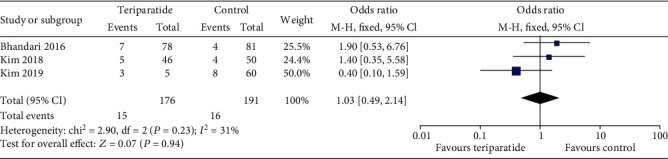
Forest plot comparing deformity in the teriparatide and control groups.

Three studies [16, 17, 20] examined deformity after fracture healing. As shown in Figure 6, no significant differences were observed between the teriparatide and control groups (OR = 1.03; 95% CI: 0.49–2.14; *P* = 0.94).

#### 3.4.4. Complications

**Figure 7:**
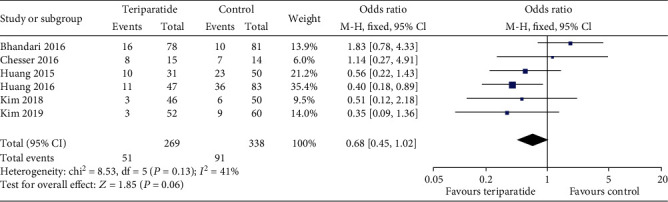
Forest plot comparing complications in the teriparatide and control groups.

All included studies assessed complications [14–17, 20, 21]. As shown in Figure 7, the aggregate results showed *I*^2^ values for heterogeneity of 41% (*P* = 0.13); thus, the fixed-effects model was used. There were no significant differences in complications (OR = 0.68; 95% CI: 0.45–1.02; *P* = 0.06) between the two groups. The results of the random-effects model showed no significant difference in mortality between the groups (OR = 0.68; 95% CI: 0.38–1.21; *P* = 0.18).

#### 3.4.5. Subsequent Fracture

**Figure 8:**
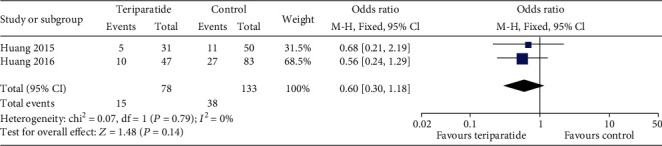
Forest plot comparing subsequent fracture between the teriparatide and control groups.

Two studies [14, 15] examined subsequent fracture in the follow-up. Comparisons between the teriparatide and control groups (Figure 8) showed no significant differences (OR = 0.60; 95% CI: 0.30–1.18; *P* = 0.14).

#### 3.4.6. Hip Function

**Figure 9:**
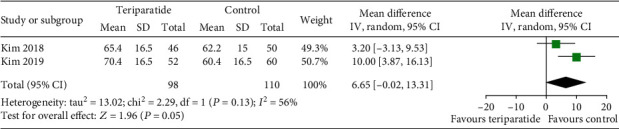
Forest plot comparing the HHS in the teriparatide and control groups.

The Harris Hip Scores (HHS) in three studies [17, 20] were compared. As shown in Figure 9, the *I*^2^ value for heterogeneity was 56% (*P* = 0.13). After excluding the possibility of clinical heterogeneity, a random-effects model showed no significant differences between the teriparatide and control groups (WMD = 6.65; 95% CI: -0.02–13.31; *P* = 0.05).

#### 3.4.7. Publication Bias

**Figure 10:**
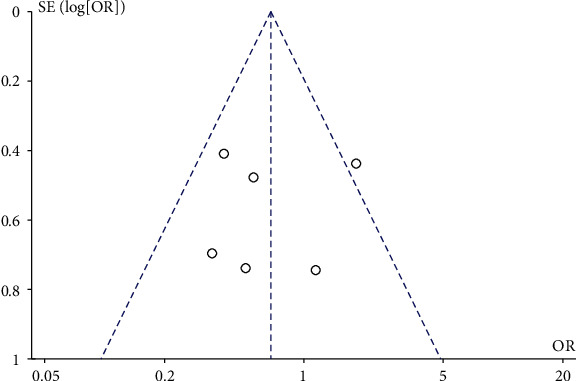
Funnel plot comparing complications in the teriparatide and control groups. The *y*-axis represents the standard error (SE) (log[OR]), while the *x*-axis represents the odds ratio (OR). The sloped lines represent the 95% confidence interval (CI) boundaries, and circles indicate the seven individual studies.

Publication bias was assessed. We chose complications for analysis. The asymmetry shown in Figure 10 suggests the potential for publication bias.

## 4. Discussion

Several studies have reported the effectiveness of teriparatide in improving bone mineral density and reducing the risk of subsequent fracture [22, 23]. However, the benefit of teriparatide in fracture healing remains controversial [24]. There has been recent increased interest in the effect of teriparatide on accelerating fracture healing [25]. Hip fractures are frequent injuries in patients with osteoporosis and are a serious burden for the individuals and their families, as well as the healthcare system [26, 27]. Thus, the use of teriparatide to accelerate hip fracture healing is of interest to orthopedic trauma surgeons.

This meta-analysis was performed to address this question. The key finding of this study was that teriparatide may have slightly accelerated the time to union but does not improve the rates of fracture union at 3 and 6 months. In addition, teriparatide did not decrease the complications, need for reoperation, mortality, rate of deformity after fracture healing, and subsequent fracture or increase hip function. A qualitative systematic review from Kim et al. [28] reported that teriparatide provided selective advantages to all fracture healing, similar to our findings of no significant difference in the healing rate. In the review of Kim et al. [28], the fracture union rate in intertrochanteric or neck fractures of the femur did not show significant differences between the groups 3, 6, and 12 months after surgery, and time to union was controversial in intertrochanteric fracture. In another qualitative review from Shin et al. [29] in 2020, they also found that the influence of teriparatide to the hip fractures was still controversial. These two important reviews used the traditional way of review with original limitation. In this present study, quantitative analysis was adopted and showed a shorter time to hip fracture union in the teriparatide group.

The primary outcomes in the present study were the time to union and the rate of fracture healing. In hip fractures, teriparatide could shorten the time to union by about 2 weeks in our results. The earlier the healing, the fewer the complications [20], especially in hip fractures in the elderly. However, teriparatide did not improve the rates of fracture union at 3 and 6 months. Biological and mechanical factors mainly influence fracture healing [30]. Teriparatide plays a biological but not mechanical role [12, 13]. Thus, teriparatide could not contribute to fracture healing by improving the percentage of fracture union; rather, it could only slightly accelerate the time to union. Among the secondary outcomes of hip fracture, teriparatide did not decrease the complications. Complications are a vital index to assess the safety of teriparatide. The complications mainly included deep and superficial wound infection, delayed union, nonunion, implant failure, reduction loss, and screw migration. The complication rates in the teriparatide and control groups in the present study were 18.96% (51/269) and 26.92% (91/338), respectively. The reoperation rates in the teriparatide and control groups were around 6.69% (17/254) and 9.26% (30/324), respectively, comparable to the 9% rate reported by Lin and Liang [31]. Moreover, teriparatide did not decrease the rates of mortality or deformity after fracture healing and did not decrease subsequent fracture or increase hip function. The above evidence seems that teriparatide plays a role in enhancing bone healing [32], without affecting other sides too much.

Our meta-analysis has several limitations. First, this study included both RCTs and observational studies. One study reported that observational studies may exaggerate the actual efficacy of teriparatide [33]. Second, slight clinical heterogeneity was observed due to differences in the daily or weekly doses of teriparatide and treatment periods between studies. The duration of treatment was too broad, from 6 weeks to 18 months. This could contribute to the heterogeneity. Third, in our meta-analysis, we have used metaregression to detect the confounding factors, but it failed because the number of included studies was less. So, we could not evaluate the possible confounding factors including reduction quality, bone mineral density, osteoporosis, type of surgery, and type of fixation device. Thus, the results should be interpreted with caution.

## 5. Conclusions

The current limited evidence did not support teriparatide improving fracture healing in hip fractures, due to study heterogeneity and various sources of biases. Further high-quality, large-sample trials are needed.

## Figures and Tables

**Table 1:** Summary of the included studies.

Study ID	Design	Diagnosis	No. of patients		Age		Primary treatment	Medical treatment		Outcome measures	Follow-up
			Teriparatide	Control	Teriparatide	Control		Teriparatide	Control		
Bhandari 2016	RCT	Femoral neck fracture	78	81	70 (50–94)	70 (50–90)	ORIF	20 *μ*g, once daily for 6 months	Placebo, identical device	Reoperation, fracture healing, pain, complications, adverse events, death, and deformity	12 months
Chesser 2016	RCT	Trochanteric fracture	15	14	80.6 ± 8.8	78.6 ± 9.3	ORIF	20 *μ*g, once daily for 6 weeks	Standard control	Death, complications, and fracture healing	3 months
Huang 2015	Retrospective study	Pertrochanteric fractures	31	50	82.3 ± 9.5	81.0 ± 8.4	ORIF	20 *μ*g, once daily for 18 months	Without receiving teriparatide	Death, reoperation, pain, complications, fracture healing, subsequent fracture, and HHS	40.1 months
Huang 2016	Retrospective study	Intertrochanteric fractures	47	83	82 ± 10	81 ± 8	ORIF	20 *μ*g, once daily for 18 months	Without receiving teriparatide	Fracture healing, complications, death, reoperation, and subsequent fracture	12 months
Kim 2018	Retrospective study	Intertrochanteric fractures	46	50	81.6 (65.8-97.9)	82.3 (67.1-99.3)	ORIF	56.5 *μ*g, weekly for 8 weeks	Without receiving teriparatide	HHS, pain, fracture healing, complications, reoperation, and deformity	6 months
Kim 2019	Retrospective study	Intertrochanteric fractures	52	60	81.4 (66.2-97.9)	80.2 (67.1-99.3)	ORIF	20 *μ*g, once daily for 2 months	Without receiving teriparatide	HHS, pain, fracture healing, complications, reoperation, and deformity	19 months

**Table 2:** Quality of the included retrospective studies.

Study ID	Selection			Comparability	Outcome		Total score
	Representativeness of the exposed cohort (maximum: ★)	Selection of the nonexposed cohort (maximum: ★)	Ascertainment of exposure (maximum: ★)	Comparability of cohorts on the basis of the design or analysis (maximum: ★★)	Assessment of outcome (maximum: ★)	Adequacy of follow-up of cohorts (maximum: ★)	
Huang 2015 [15]		★	★	★★	★		5
Huang 2016 [14]		★	★	★★		★	5
Kim 2018 [17]		★	★	★★		★	5
Kim 2019 [20]		★	★	★★	★	★	6

## Abbreviations

RCT: Randomized controlled trials
WMD: Weighted mean difference
CI: Confidence interval
OR: Odds ratio
ORIF: Open reduction and internal fixation
NOS: Newcastle-Ottawa Scale
M-H: Mantel-Haenszel
I-V: Inverse variance
HHS: Harris Hip Scores.
